# Exposure to a dog elicits different cardiovascular and behavioral effects in pregnant and lactating goats

**DOI:** 10.1186/1751-0147-53-60

**Published:** 2011-11-16

**Authors:** Kerstin Olsson, Eva Hydbring-Sandberg

**Affiliations:** 1 Department of Anatomy, Physiology, and Biochemistry, Faculty of Veterinary Medicine and Animal Science, Swedish University of Agricultural Sciences, Box 7011, SE-750 07 Uppsala, Sweden

**Keywords:** activity, arterial blood pressure, behavior, cortisol, heart rate

## Abstract

**Background:**

Heart rate and plasma cortisol concentration are often used in evaluation of physiological reactions to stress and fear, but arterial blood pressure is rarely measured in farm animals. Goats are prey animals and can be expected to react strongly to a predator, especially when they have kids. We hypothesized that exposure to a dog elicits a flight response during pregnancy and a fight response when goats have kids to defend. Arterial blood pressure and heart rate should increase in both these cases, due to a synchronized discharge of the sympathetic nervous system.

**Methods:**

Seven goats were exposed to a dog for 15 minutes at 12 ± 3 days before, and again at 10 ± 1 days after, parturition. Arterial blood pressure, heart rate, and activity were registered by telemetry. Behavioral data were collected during 5 minute sessions, followed by blood samples obtained via intrajugular catheters. Plasma cortisol concentration was analyzed by radioimmunoassay.

**Results:**

At the appearance of the dog, the mean arterial blood pressure of the goats increased from 90 ± 8 to 111 ± 8 mmHg (p < 0.001) during pregnancy and from 96 ± 8 to 108 ± 8 mmHg during lactation (p < 0.001). Heart rate did not change at dog exposure during lactation, but increased from 117 ± 6 to 126 ± 10 beats/min (p < 0.01) during pregnancy. Dog exposure resulted in plasma cortisol concentration increasing from 17 ± 1 to 43 ± 7 nmol/l (p < 0.01) during pregnancy and from 21 ± 1 to 49 ± 6 nmol/l (p < 0.01) during lactation. In response to the dog, goats vocalized at a higher frequency and started to ruminate later during lactation compared to pregnancy.

**Conclusions:**

When goats were exposed to a dog during pregnancy, their heart rate, blood pressure, and plasma cortisol increased, in contrast to lactation when only their blood pressure and plasma cortisol increased. However, when they were lactating, goats vocalized more and started to ruminate later compared to when they were pregnant.

## Background

In the classic "fight or flight" response to a threatening event, the sympathetic nervous system and the hypothalamic-pituitary-adrenal (HPA) axis are activated [1,2]. If the animal runs away, the arterial blood pressure and heart rate increase simultaneously, but in other situations, the animal stands still and even "freezes." When the latter occurs, an elevated arterial blood pressure may be accompanied by bradycardia [3]. The response of the animal is dependent not only on the stimulus, but also on the ability of the individual animal to cope with the situation [4]. Consequently, it is of value to measure both the arterial blood pressure and the heart rate simultaneously when evaluating the cardiovascular response to different situations. Techniques for telemetrically measuring heart rate in freely moving animals have been used for many years, but methods for measuring blood pressure have been more difficult to develop. The non-invasive cuff method may disturb some animals, while conventional, invasive methods involve catheterization of an artery and necessitate that the animal is restrained [5]. These difficulties were overcome when a telemetric method for blood pressure measurements was developed in rats [6] and modified for use in freely moving goats [5].

Goats are prey animals and can be expected to react strongly to a predator, especially when they have kids [7,8]. This study recorded behavior during simultaneous registrations of blood pressure and heart rate in goats exposed to a predator. The same goats were studied during pregnancy and again during lactation. Plasma cortisol concentration was analyzed to determine if the HPA axis was engaged [9].

We hypothesized that exposure to a dog elicits a flight response during pregnancy and a fight response during lactation, when the goats have kids to defend, and that arterial blood pressure and heart rate increase on both occasions.

## Methods

### Animals

Seven 2-4-year-old Swedish domestic landrace goats (*Capra hircus*) weighing 47 ± 2 kg (during lactation) were studied. Goats and kids were routinely kept together with the entire herd in a large, indoor pen. The goats were fed at 07:00 and 15:00 and had free access to water and mineral licks. Room temperature was kept at 17 ± 1°C.

The goats were accustomed to walk to another room with individual cages (1.20 m wide × 1.50 m long × 1.30 m high) along the walls and an open floor area measuring 4.0 m × 4.0 m in the middle. Hay and water were available in the cages. The kids (six twins and one singleton) accompanied their mothers to the cages.

The care of the animals and the experimental design were approved by the Local Ethical Committee in Uppsala, Sweden.

### Telemetry

The goats had previously implanted telemetric devices (Data Sciences, Inc., St Paul, MN, USA) for registering arterial blood pressure, heart rate, and activity. In brief, under full anesthesia, the transmitter body had been placed subcutaneously on the lateral side of the neck, and the fluid-filled catheter had been tunneled subcutaneously to the superficial temporal artery and, further, into the carotid artery [5]. During monitoring, blood pressure and heart rate signals were transmitted to a receiver placed centrally above each cage, and the signals were digitized. Data was collected for 10 seconds every 2 minutes from each animal. Activity is a relative measure of the signal quality between the transmitter and the receiver when the animal moves and is sampled continuously. A high activity means that the animal was active, but it does not relate to absolute distance moved or to spatial position. Activity is expressed as units proportional to the degree of change in the signal strength, given as units per minute. The units are arbitrary but consistent throughout. The software package used was DataQuest IV (Data Sciences, Inc.).

### Experimental design

The goats were exposed to the dog for 15 minutes at 12 ± 3 days before parturition and again at 10 ± 1 days after parturition. Telemetric registrations started when the goats arrived at the cages. At 07:30, a local anesthetic ointment (EMLA^®^, AstraZeneca, Södertälje, Sweden) was applied to the shaved skin covering the jugular veins. About 60 minutes later, a catheter (Secalon^®^, Ohmeda, Swindon, UK) was inserted into one of the jugular veins in a maximum of four goats on one experimental day.

At 10:30, a German Shepherd Dog, trained in obedience, entered the room and walked at heel with his owner along the cages for 5 minutes. Thereafter, the dog played with a toy in the middle of the room for 5 minutes. Finally, the owner and the dog walked to each cage, the dog sat down, and the owner offered the goat a piece of apple to test if the goat dared to come forward. If the goat did not take the piece of fruit, the owner dropped it in the cage. This procedure was repeated with the next goat. Altogether, distribution of apple pieces took 5 minutes.

### Blood samples

**Figure 1:**
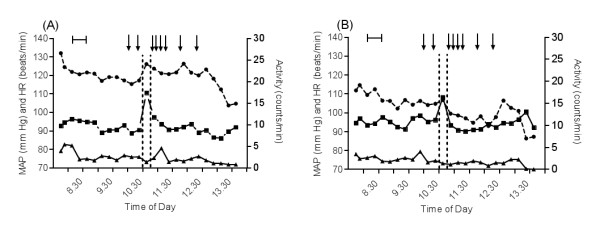
**Cardiovascular responses of goats to the appearance of a dog**. Mean arterial blood pressure (MAP; squares), heart rate (HR; circles), and activity (triangles) in seven goats exposed to a dog between 10:30 and 10:45 (vertical, hatched lines), first during pregnancy (A) and later during lactation (B). Line within brackets: intrajugular catheters inserted. Arrows: blood sampling. Values are presented as least square means (LSM) of data collected every 2 minutes in each goat, and summarized over 15 minutes. Standard error (SE) was: 8 (MAP), 6 (HR), and 0.8 (activity). In pregnant goats, MAP and HR increased at dog presence (p < 0.01-0.001 vs. 15 minutes before introduction of the dog) but there was no change in activity. In lactating goats, MAP increased at dog presence (p < 0.001 vs. 15 minutes before introduction of the dog), but HR and activity did not change.

The goats were lightly restrained while the blood was withdrawn. Blood samples were taken at 10:00 and 10:20. No sample was taken while the dog was in the room, but the third sample was withdrawn immediately thereafter, i.e., at 10:45, and again at 10:55, 11:05, 11:15, 11:45, and 12:15 (Figure 1A and 1B, arrows). The samples (10 ml each) were collected into tubes containing K_3_-ethylenediaminetetra-acetic acid and centrifuged at + 4°C for 20 minutes at 1500 g and stored at -70°C until assayed. Cortisol concentration was analyzed using a radioimmunoassay (Coat-A-Count; Diagnostics Product Corporation, Los Angeles, CA, USA) validated for caprine plasma. The lower detection limit was 1.10 nmol/l, the interassay coefficient of variance was < 1.6% (at 34 nmol/l), and the intraassay coefficient of variance was < 10% (values between 6 and 1380 nmol/l).

### Behavior

The behavior of the goats was registered simultaneously by two observers (each of them observing one or two goats). The behavioral data were collected during 5 minute observation sessions, starting at 09:55 and at 10:15. The first sessions took place before the dog entered, three sessions occurred during dog presence, and thereafter the sessions started again at 10:50, 11:00, 11:10, 11:40, and 12:10. During each session, 15 sample points (at 20 second intervals) were collected using an electronic timer. At each sample point, the activities walking, standing, lying down, climbing, stamping, eating, drinking, ruminating, and vocalizing (see below for Definitions) were recorded on a score sheet. In addition, all sessions were videotaped. If the goats took the piece of apple, this was noted, as was the time at which this took place (see Experimental design).

### Definitions

*Walking*: moving forward, lifting one foot after the other. This occurred rarely and has therefore been summarized together with *standing*.

*Standing: *standing with all four feet on the floor.

*Lying down*: lying with all four legs under the body and the head either held up or resting on the ground.

*Climbing: *putting one or two front legs on the horizontal bars of the cages. The frequency was counted continuously during each 5 minute session.

*Stamping*: abruptly lifting and putting down one front leg. The frequency was counted continuously during each 5 minute session.

*Ruminating: *regurgitating and chewing previously swallowed food.

*Vocalizing*: bleating. The frequency was counted continuously during each 5 minute session (145 or more bleats per 5 minute session = 100%).

*Eating: *taking something edible into the mouth.

*Drinking*: putting the mouth in a bucket of water (occurred rarely).

### Statistical analyses

The data were analyzed with SAS^® ^software (SAS Institute, Inc., Cary, NC, USA). The repeated measurement analysis of variance (using the MIXED procedure) was applied to the various parameters. The statistical analysis included the fixed effects of mean arterial blood pressure, heart rate, activity, blood sample, behavior, and reproductive period, and the random effect of goat.

Telemetry data were arranged in blocks of 15 minutes. The behavioral data were logarithmically transformed before the analysis. Values are presented as means ± standard error of the means (SEM) for behavior and cortisol data, and as least square means (LSM) and standard error (SE) of LSM for mean arterial blood pressure, heart rate, and activity data. Means were estimated and pairwise tests of significance were performed for the differences between the estimated means. Bonferroni adjustment was used to limit the risk for false mass significance. The significance level was set at p ≤ 0.05.

## Results

### Blood pressure, heart rate, and activity

As illustrated in Figure 1A, mean arterial blood pressure increased from 90 ± 8 mmHg to 111 ± 8 mmHg when the dog was visible to the pregnant goats (p < 0.001 vs. all values before and after). Blood pressure decreased as soon as the dog left the room. Heart rate was 117 ± 6 beats/min just before the dog arrived. It increased to 126 ± 6 beats/min (p < 0.01) and then remained elevated for about 2 more hours (Figure 1A). The activity level was 2.5 ± 0.8 counts/min before, and 1.4 ± 0.8 counts/min during, dog presence (ns) (Figure 1A).

In Figure 1B, the changes in mean arterial blood pressure, heart rate, and activity in lactating goats are illustrated. Blood pressure increased from 96 ± 8 to 108 ± 8 mmHg following the introduction of the dog (p < 0.001 vs. all values before and after). Heart rate was 105 ± 6 beats/min before the dog entered, and 107 ± 6 beats/min while the dog was present (ns). The activity was 2.0 ± 0.8 counts/min before, and 1.0 ± 0.8 counts/min during, dog presence (ns).

Overall comparison of blood pressure, heart rate, and activity between pregnancy and lactation revealed that the blood pressure was lowest during pregnancy (93 ± 8 vs. 95 ± 8 mmHg, p < 0.001), whereas the heart rate was highest during pregnancy (121 ± 6 vs. 104 ± 6 beats/min, p < 0.001). The goats were most active during pregnancy (2.4 ± 0.6 vs. 2.0 ± 0.6 counts/min, p < 0.05).

### Plasma cortisol concentration

**Figure 2:**
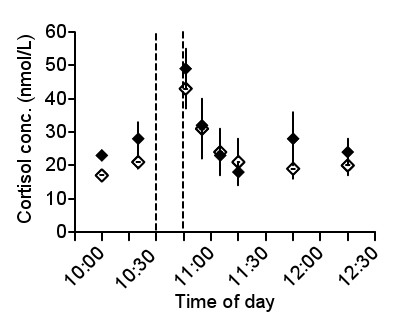
**Plasma cortisol response in goats to the appearance of a dog**. Plasma cortisol concentration in seven goats exposed to a dog between 10:30 and 10:45 (vertical, hatched lines), first during pregnancy (open symbols) and later during lactation (filled symbols). Data are presented as means ± standard error of the mean (SEM). Plasma cortisol concentration was elevated in the sample withdrawn at 10:45 compared to the sample taken at 10:00 (p < 0.05) during both pregnancy and lactation, but did not differ from the samples obtained at 10:20.

The plasma cortisol concentration was elevated in the sample taken immediately after the dog left (Figure 2). In pregnant animals, it increased from 17 ± 1 nmol/l at 10:00 to 43 ± 6 nmol/l at 10:45 (p < 0.01). In lactating animals, it increased from 21 ± 1 nmol/l to 49 ± 6 nmol/l (p < 0.01). The difference between the samples taken at 10:20 and 10:45 was not, however, significant.

### Behavior

**Figure 3:**
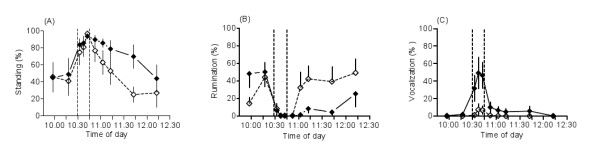
**Behavioral responses of goats to the appearance of a dog**. Standing (A), rumination (B), and vocalization (C) in the same seven goats during pregnancy (open rhombs) and lactation (black rhombs). The goats were exposed to a dog between 10:30 and 10:45 (vertical, hatched lines). Data are given as means ± standard error of the mean (SEM). During dog presence, frequency of standing increased (p < 0.05 vs. the observations before) and rumination decreased (p < 0.05 vs. 10:15) during both pregnancy and lactation. Rumination was resumed earlier during pregnancy compared to lactation (p < 0.05). In lactating goats, vocalization increased (p < 0.001 during dog exposure vs. both before exposure to the dog and pregnancy) and continued after the dog left (p < 0.01 vs. pregnancy).

Before the dog came in, about 50% of the goats were lying down and had started to ruminate (Figure 3A and 3B), but most of them were standing (p < 0.05 vs. before the dog) and did not ruminate (p < 0.01 vs. before the dog) during dog presence. The goats remained standing after the dog left (ns between pregnancy and lactation), but the goats started to ruminate faster during pregnancy than during lactation (p < 0.05).

Infrequent bleats were heard from pregnant goats in the presence of the dog (ns), but lactating goats started to vocalize as soon as they caught sight of the dog, and vocalized intensively while the dog was present (p < 0.001 vs. both before the dog and pregnancy). Lactating goats continued to vocalize after the dog had left (p < 0.01, pregnancy vs. lactation).

The goats climbed or stamped a few times during the experiments. Before the dog arrived, pregnant goats did not climb, but lactating goats climbed 3 ± 2% (ns) of the time. When the dog was present, only pregnant goats climbed, at a rate of 8 ± 2% (p < 0.01 vs. before the dog; p < 0.05 vs. lactation). Pregnant animals stamped 6 ± 2% (p < 0.01 vs. before the dog), while lactating goats stamped 3 ± 2%, of the time (ns). After the dog had left, pregnant goats climbed 2 ± 2% of the time between 10:50 and 10:55 (ns).

During pregnancy, four of the goats took the piece of apple; three goats did not touch it. During lactation, six of the goats took the apple piece at once, and one of them bit the hand of the owner before taking the apple.

Before the dog came in, the kids were lying down most of the time. When the dog arrived, all the kids except the singleton and one twin kid stood up watching the dog. Two goats were suckled before dog exposure and one goat after.

## Discussion

Arterial blood pressure and heart rate increased simultaneously when pregnant goats saw the dog. In lactating goats, blood pressure increased but the heart rate did not change, although the goats appeared as alarmed during lactation as they did during pregnancy.

Many ruminants are pregnant or lactating most of the year and in addition, farm animals are kept for high production. One difference between pregnant and lactating animals is that the baroreceptor reflex is attenuated during pregnancy and returns to previous levels during lactation in most animals including goats [10,11]. Consequently, the heart rate and blood pressure could increase simultaneously in pregnant goats, but when the blood pressure increased during lactation the baroreceptor reflex may have prevented a rise in heart rate.

Goats are curious animals and do not easily panic if something unexpected happens [7]. The goats studied here were born in our animal house and were used to the people handling them and to experimental procedures. This may explain why they became alert, but did not try to jump over the cage walls and run away when they saw the dog for the first time. Nevertheless, they were alarmed, as shown by the elevated blood pressure, heart rate, and plasma cortisol concentration.

After a few weeks, when the goats saw the dog for a second time, they may have recognized the situation and reacted differently for that reason. In another study, the first time that sheep were exposed to a sudden event they showed a startle response and tachycardia, but the response was smaller when the experiments were repeated [12]. It may be that the goats remembered that nothing happened to them the previous time, and this could explain the unchanged heart rate. Their alerted behavior, as well as the rise in blood pressure and plasma cortisol concentration, was in contradiction to this.

The activity counts were low when the dog was present, which shows that the signal strength from the sensor body on the neck of the goats varied only a little, i.e., the goats stood nearly still. This was supported by direct observations. During pregnancy, the goats climbed and stamped, but did not run around in the cage; during lactation, they stamped a few times but otherwise stood watching the dog. Pregnant goats uttered a few bleats, but lactating goats vocalized intensively with a high-pitched noise that sounded like a sneeze. This noise seems to be typical of alarmed goats [13].

When animals stand still in response to a threatening event, activity in the sympathetic nervous system may increase blood pressure and redirect the blood to muscles and the brain to prepare the animal for fight or flight at the same time as activation of the parasympathetic innervation of the heart prevents a rise in heart rate [3].

In lactating rats, aggressive behavior has been observed simultaneously with an attenuated responsiveness of the sympathetic nervous system as well as the HPA axis [14]. In this study, one of the goats became aggressive towards the dog and owner when it had the kids in the cage, but there was no change in heart rate during lactation. However, both blood pressure and cortisol increased and the responses did not differ between pregnancy and lactation.

It could be questioned how much the dog disturbed the goats in relation to other events that took place during the day. Eating is accompanied by a marked rise in heart rate in goats fed in the morning and afternoon [15], and the heart rate did not increase above eating levels during the presence of the dog. The blood pressure was markedly elevated in the presence of the dog, but the rise was relatively small during lactation. This was probably due to a generally elevated blood pressure during lactation compared to pregnancy in this study compared to previous reports [15,16]. In those previous studies, goats were separated from their kids soon after birth, which is common in dairy goat farms [17]. It appeared that the goats were more tense when they were accompanied by their kids during the experiment.

## Conclusions

When goats were exposed to a dog during pregnancy, their heart rate, blood pressure, and plasma cortisol increased, in contrast to lactation when only their blood pressure and plasma cortisol increased. However, when they were lactating, goats vocalized more and started to ruminate later compared to when they were pregnant.

## Competing interests

The authors declare that they have no competing interests.

## Authors' contributions

EHS participated in the design and coordination of the study, in particular with regard to behavior, and drafted the manuscript. KO participated in the design of the study, performed the statistical analysis and discussed the draft of the manuscript. Both authors approved the final manuscript.
